# A comprehensive investigation of energy consumption and carbon emissions for a new type of cold mix asphalt technology

**DOI:** 10.1038/s41598-026-55418-8

**Published:** 2026-06-03

**Authors:** Qian Guo, Zhuan Wang

**Affiliations:** 1 Department of Architectural Engineering, Guizhou Communications Polytechnic University, Guiyang, 551400 China; 2 Key Laboratory of High Performance Repair Materials, Guiyang, 551400 China

**Keywords:** Environmental sustainability, Greenhouse gases, Pavement materials, Sustainable infrastructure, Climate sciences, Energy science and technology, Engineering, Environmental sciences

## Abstract

Cold mix asphalt (CMA) offers promising potential for reducing the energy consumption and greenhouse gas (GHG) emissions associated with traditional hot mix asphalt (HMA). This study presents a comprehensive investigation of the energy consumption and carbon emissions associated with a newly developed CMA technology (New-CMA) incorporating a hydroxylated cycloalkane-based polymer additive, which improves workability at ambient temperatures. Energy consumption and carbon emissions were quantified across the whole production stages, using energy consumption method and emission factor method recommended by the Intergovernmental Panel on Climate Change (IPCC). The results demonstrate that the New-CMA achieves a 48% reduction in total energy consumption and a 40% reduction in carbon emissions compared to conventional HMA. Significant reductions were observed particularly in the mixture production stage, where energy use and carbon emissions decreased by 97.6% and 93.6%, respectively, due to the elimination of aggregate heating. Compared to traditional CMA, the New-CMA shows reductions of 9% in energy use and 10% in carbon emissions. These improvements are primarily attributed to the elimination of aggregate heating, lower mixing temperatures, and enhanced mixing efficiency. The technology is particularly suitable for preventive maintenance and structural layers in low-to-medium traffic roads. This study provides a comprehensive environmental evaluation and offers guidance for promoting sustainable pavement practices in urban construction.

## Introduction

Global warming remains one of the most pressing global challenges^1,2^. To address this issue, industries across various sectors are increasingly adopting sustainable practices^3^. Similarly, the highway industry is also adopting several measures to reduce its carbon footprint^4^. Asphalt mixtures, the primary material in road construction^5,6^, have drawn considerable attention due to their significant energy consumption and carbon emissions during both production and use. CMA is considered as a potential low-carbon alternative due to its ambient temperature construction characteristics^7–11^.

The conventional CMA is defined as an asphalt mixture produced by mixing emulsified bitumen, cutback, or foamed bitumen with un-heated aggregates^4^. CMA offers several key benefits, including its cost-efficiency, environmental friendliness, reduced emissions, and ready availability. However, it also has certain drawbacks, such as lower initial strength in the early stages, higher levels of voids, and greater susceptibility to moisture^12^. From an environmental sustainability perspective, CMA stands out for its friendliness: by avoiding high-temperature production processes, it significantly cuts down on greenhouse gas emissions compared to HMA, aligning with sustainable construction practices and reducing the carbon footprint of road projects. If asphalt mixtures can be produced and processed at ambient temperatures—while still maintaining comparable or even better engineering performance—this would yield significant economic and environmental benefits. For these reasons, developing innovative construction technologies to serve as a viable alternative to HMA is of critical importance^7,13^.

Several manufacturing techniques have been developed for cold mix asphalt to enable ambient-temperature production. Emulsified asphalt is the most common approach, where bitumen is dispersed in water with the aid of emulsifiers, allowing mixing with moist aggregates at ambient temperatures^8,12^. Foamed bitumen, produced by injecting small amounts of cold water and air into hot bitumen, creates a foam that expands and coats aggregates without heating^7,10^. More recently, chemical additives have been explored to modify binder rheology and improve workability at lower temperatures. For instance, polymer-modified emulsions have been shown to enhance adhesion and cohesion properties, leading to improved early strength and moisture resistance^4^. Bio-based additives and reactive diluents have also demonstrated potential for reducing binder viscosity without compromising long-term performance^11^. Despite these advances, the energy consumption and carbon emission implications of such modified CMA systems have received limited attention. Most existing studies focus primarily on mechanical performance and workability^12^, while systematic quantification of environmental impacts—particularly across multiple stages of production and construction—remains scarce^14,15^. This gap is particularly relevant because additive formulations directly influence not only mixing and compaction temperatures but also the overall process energy demand. Additives that enable the use of unheated aggregates or reduce mixing energy may yield significant environmental benefits that have not been fully captured in previous studies. The hydroxylated cycloalkane-based polymer additive employed in this study is designed to address this gap by enabling effective coating of unheated aggregates while promoting rapid early strength development. A comprehensive evaluation of both the technical and environmental performance of this novel CMA technology is therefore needed to support its broader adoption in sustainable pavement construction.

To address the need for comprehensive environmental evaluation of CMA technologies, various methodological approaches have been employed in previous studies. Studies on carbon emissions in pavement construction typically adopt methodologies such as LCA^16–21^, experimental studies, modeling and data analysis. Studies have found that the production and utilization phases of asphalt pavements have significant environmental impacts and are subject to a variety of factors. Many studies have adopted the LCA methodology to evaluate the environmental performance of asphalt pavements across their full life cycle—from raw material extraction to final disposal. These studies have established systematic frameworks based on defined system boundaries, data collection, inventory analysis, and environmental impact assessment, enabling the quantification of key indicators such as energy consumption and carbon emissions. Ferrotti et al.^22^ conducted comparative experiments by laying HMA and warm mix asphalt (WMA) on-site on a highway in Italy to measure energy consumption and emission data at different stages. These experiments generated primary data, thereby improving the credibility of the findings. Liu et al.^23^ developed a carbon dioxide emission model for the asphalt mixing process to analyze the relationship between factors such as mixing time, mixer capacity and carbon emissions; Santos et al.^24^ developed an energy balance model to calculate the thermal energy required for asphalt mixture production and emissions; Gruber & Hofko^25^ used material flow software to estimate GHG emissions at each stage, which helped reveal the underlying environmental mechanisms. Schönauer et al.^1^ processed and analyzed two years of production data from a batch asphalt mixing plant, and used linear discriminant analysis to investigate the effects of factors such as production scale and asphalt mixture temperature on energy consumption; Sollazzo et al.^26^ used sensitivity analyses to investigate the environmental impacts of variables such as production rate and fuel consumption. The data analysis has unearthed the patterns behind the data and provided strong support for the research conclusions.

While the above studies have provided valuable insights, several limitations remain in existing research. Some studies rely heavily on secondary sources, which compromises the accuracy and reliability of the results, e.g., most production and technical data in some LCA studies are sourced from generic databases, which may not accurately reflect real-world conditions^25,26^. From a methodological perspective, there are variations in system boundary definitions and selection of impact assessment methods in the LCA methodology, leading to a lack of comparability of the results. For example, different studies have different divisions and trade-offs between road life cycle stages, and the methods and data sources used in calculating energy consumption and emissions are inconsistent^24,27^. In terms of the limitations of experimental research, experimental studies are mostly conducted under specific conditions with limited experimental scale and scope, limiting the broader applicability of the findings. For example, some experiments on WMA technology are only conducted in specific laboratories or small-scale construction sites, making it difficult to generalize to a wider range of practical application scenarios^1,28^.

In contrast, the present study utilizes primary data collected from field operations and actual mixing plant production records to ensure that critical stages—particularly mixture production—are accurately represented. Based on energy consumption data and the carbon emission factor method recommended by the IPCC, a quantitative method is developed to evaluate the energy consumption and carbon emissions of the New-CMA. The analysis is conducted within a system boundary that includes material production, material transportation, mixture production, mixture transportation, and pavement construction. A comparative analysis is performed between conventional HMA, traditional CMA, and the New-CMA. The specific objectives of this study are as follows:

（1）To establish a quantitative model for assessing energy consumption and carbon emissions across five key stages of asphalt pavement construction: raw material production, material transportation, mixture production, mixture transportation, and pavement construction.

（2）To evaluate and compare the environmental performance of a newly developed cold mix asphalt (New-CMA) against conventional hot mix asphalt (HMA) and traditional cold mix asphalt (CMA).

（3）To identify the key stages contributing to energy consumption and carbon emissions in the production and construction processes of different asphalt mixtures, thereby providing insights for targeted environmental impact mitigation.

（4）To provide theoretical support for the development and application of low-carbon pavement materials in sustainable road infrastructure.

By quantifying energy use and emissions across all key production stages, the study reveals the significant energy-saving and emission-reduction potential of CMA technologies. The findings provide a theoretical foundation for the research, development, and wider application of low-carbon pavement materials in sustainable road construction.

## Materials and methods

### Study area and data collection

The field investigation was conducted at a batch-type asphalt mixing plant located in Guizhou Province, China. The region has a typical subtropical plateau monsoon climate with an average annual temperature of 14–16°C. Summer temperatures average 21–24°C, while winter temperatures average 5–8°C, providing a representative setting for asphalt mixture production under varying ambient conditions. Primary data were collected through on-site measurements, equipment monitoring, and operational records over a production period of three months (June to August 2023).

### Materials

#### Description of the new-CMA

**Fig. 1:**
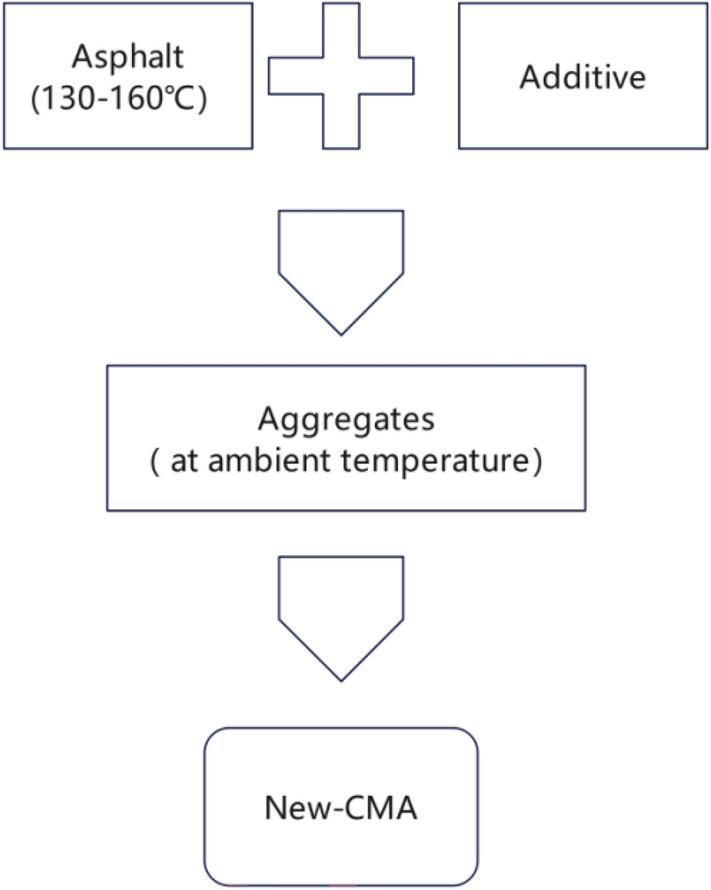
The production process of the New-CMA.

The New-CMA employed in this study is produced by blending heated asphalt at 130–160 °C with a specific dosage of additive while keeping the aggregates unheated (Fig. 1). This approach eliminates the need for aggregate heating, significantly reducing energy consumption during the mixing process.

The material properties of the New-CMA are as follows:

1. Aggregates: Limestone aggregates with a continuous gradation conforming to AC-13 specification (Chinese standard JTG F40-2004). Bulk specific density: ≈2.72 g/cm3; water absorption: < 1.0%.
2. Asphalt: 70-pen base asphalt binder. Penetration (25°C): 66 dmm; softening point: 53°C; ductility (15°C): > 100 cm.
3. Additive: A hydroxylated cycloalkane-based polymer additive was used at 12% by weight of asphalt. Its primary function is to reduce initial viscosity during mixing and promote later strength development through chemical restructuring.

Detailed characterization of the additive’s effects on binder rheology and mixture mechanical performance will be presented in a separate follow-up publication. The additive production stage is not quantitatively included in this study due to proprietary data limitations.

#### Mixing process

The mixing process for New-CMA (Fig. 1) consists of four steps:

1. Asphalt heating: Asphalt binder is heated to 130–160 °C in an electrical heating tank.
2. Additive incorporation: The additive (12% by weight of asphalt) is added to the heated binder and mechanically stirred for 1–2 min.
3. Mixing: The modified binder is mixed with unheated aggregates in a batch-type mixing unit for 60–90 s.
4. Output: The mixture is discharged at ambient temperature and ready for paving.

No aggregate heating is applied at any stage.

#### Production process comparison

To further clarify the distinctions among HMA, conventional CMA, and New-CMA, Table 1 provides a comparative summary of their production processes. As shown in Table 1, the key distinction between HMA and New-CMA lies in the elimination of aggregate heating. In HMA production, both the binder and aggregates must be heated to high temperatures (150–180°C) to achieve adequate workability and aggregate coating. In contrast, New-CMA requires only the binder to be heated (130–160°C), while aggregates remain at ambient temperature (15–25°C). Conventional CMA, which uses emulsified or foamed bitumen, requires no heating of either binder or aggregates but may have different performance characteristics.

**Table 1:** Comparison of heating requirements for HMA, conventional CMA, and New-CMA.

Parameter	HMA	Conventional CMA	New-CMA
Asphalt binder heating	150–180 °C	Not heated (emulsion or foam)	130–160 °C
Aggregate heating	150–180 °C	Not heated	Not heated
Additive type	None typically	Emulsifiers	Hydroxylated cycloalkane-based polymer
Mixing temperature	150–180 °C	Ambient (15–25 °C)	Ambient (15–25 °C)

### Methods

#### System boundary definition

**Fig. 2:**
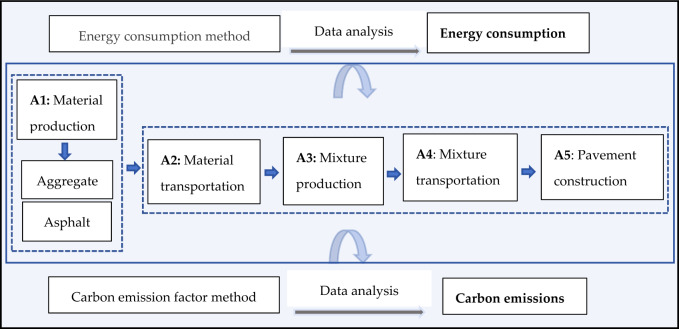
The evaluation system of energy consumption and carbon emission for asphalt mixture.

This study adopts a cradle-to-site system boundary, covering all stages from raw material extraction to the completion of pavement construction. As illustrated in Fig. 2, the system boundary includes five key stages:

A1: Material production.

This stage encompasses the extraction, processing, and manufacturing of all raw materials.

A2:Material transportation.

This stage covers the transportation of the raw materials from their production or processing facilities to the asphalt mixing plant.

A3: Mixture production.

This is the core stage where the raw materials are blended into the final asphalt mixture. This stage involves the operation of the mixing plant.

A4: Mixture transportation.

This stage involves transporting the finished asphalt mixture from the mixing plant to the construction site.

A5: Pavement construction.

The pavement construction stage primarily involves the operation of pavers and rollers.

This study first decomposes the asphalt pavement construction process into different sequential stages. For energy consumption analysis, the energy consumption method is adopted. After parameter selection, the stage—specific and the overall energy consumption is calculated. For carbon emissions analysis, the carbon emission factor method is used. Through parameter selection, the carbon emissions of each stage and the whole project are quantified. The use phase, maintenance activities, and end-of-life treatment are excluded from the system boundary. This boundary definition is justified by the primary focus of this study on production and construction stages, as well as the lack of reliable long-term performance data for the New-CMA technology.

#### Functional unit

The functional unit of this study is defined as 1000 m^3^ of asphalt mixture. This volume-based unit was selected because pavement construction is typically planned and executed based on volumetric quantities (e.g., cubic meters of pavement layer). Using volume as the functional unit facilitates direct application of the results to construction projects, where the required quantity of asphalt mixture is specified in cubic meters. To enable comparison with other studies that use mass-based functional units (e.g., per ton of mixture), the results are also normalized to per ton of asphalt mixture in the comparative analysis. This dual presentation ensures both practical applicability and comparability with existing literature.

#### Calculation formula

 (1) Energy consumption is calculated as the aggregate consumption of different energy sources at each stage, with diesel and electricity being the primary energy sources in this study. Among them, diesel energy consumption is diesel consumption multiplied by diesel combustion calorific value, electric power energy consumption is electricity multiplied by electric power energy calorific value, and the total energy consumption is calculated as the sum of all stage-specific energy inputs^29^.

(2) According to the IPCC method^29^, carbon emissions are calculated using the carbon emission factor method, and the results are expressed in carbon dioxide equivalent (kgCO_2_e). The calculation formula is presented in Eq. (1):

1 $${\mathrm{G}}_{{\mathrm{i}}} = Q_{i} \times {\mathrm{EF}}_{{\mathrm{i}}} ,$$

where G_i_ is the total carbon emission (kgCO_2_e), *Q*_*i*_ is the activity intensity value (kg, kw·h, etc.) and EF_i_ is the carbon emission factor [kgCO_2_e/t, kgCO_2_e/kg, kgCO_2_e/(kw·h)], etc.

The total carbon emissions are the superposition of the carbon emissions of each process and the calculation formula is presented in Eq. (2):

2 $${\text{G }} = {\text{ G}}_{{1}} + {\mathrm{G}}_{{2}} + {\mathrm{G}}_{{3}} ,$$

where G is the total carbon emission (kgCO_2_e), G_1_ is the carbon emission in the material production and transportation stage (kgCO_2_e), G_2_ is the carbon emission in the mix production and transportation stage (kgCO_2_e), and G_3_ is the carbon emission in the pavement construction stage (kgCO_2_e).

#### Data analysis

##### Material production

The energy consumption and carbon emissions in the material production stage, which is mainly generated by the energy consumed in the production of asphalt and aggregates, are referenced by the European Asphalt Association and the Chinese Life Cycle Database (CLCD)^30^. The parameters selected are shown in Table 2.

**Table 2:** Parameter selection of asphalt and aggregates.

Types	Energy consumption (MJ/t)	CO_2_ emissions (kgCO_2_e/t)
Asphalt	2830.76	189.12
Aggregates	31.82	2.43

##### Material transportation

Material transportation energy consumption refers to the energy consumption of materials transported from the processing plant to the mixing plant. The energy consumption of different transportation modes and distances is different, with 30t diesel truck road transportation distance of 50km as the standard referenced by the CLCD. The selected parameters are shown in Table 3.

**Table 3:** Parameter selection of material transportation.

Types	Energy consumption (MJ/t)	Carbon emissions (kgCO_2_e/t)
Material transportation (50km)	40.20	3.74

##### Mixture production

The energy use of mixture production primarily involves the loader and mixing tower operations, in which the energy consumption of the mixing tower is mainly used for the heating of asphalt, aggregate and mixing of various materials. In this study, it is mainly electrical power consumption, and the selected parameters are shown in Table 4. The electricity emission factor is based on the 2012 average carbon emission data for China’s regional power grids, published by the National Center for Climate Strategy.

**Table 4:** Parameter selection of electrical power.

Types	Electrical power calorific value [MJ/(kw·h)]	Carbon emission factor [kgCO_2_e/(kw·h)]
Electrical power	3.60	0.5271

##### Mixture transportation and pavement construction

Transportation of the mixtureis carried out by tipper trucks within 15t, which is mainly diesel fuel consumption. The machines in the construction stage are pavers and rollers, and the energy consumption and carbon emission calculations are based on diesel consumption. The parameters are selected as in Table 5, where the diesel carbon emission factors refer to the greenhouse gas emission factors provided by the IPCC database.

**Table 5:** Parameter selection of diesel fuel.

Types	Calorific value of diesel fuel (MJ/kg)	Carbon emission factor (kgCO_2_e/kg)
Diesel fuel	42.65	3.14

## Data and calculation

### Material production and transportation stage

**Fig. 3:**
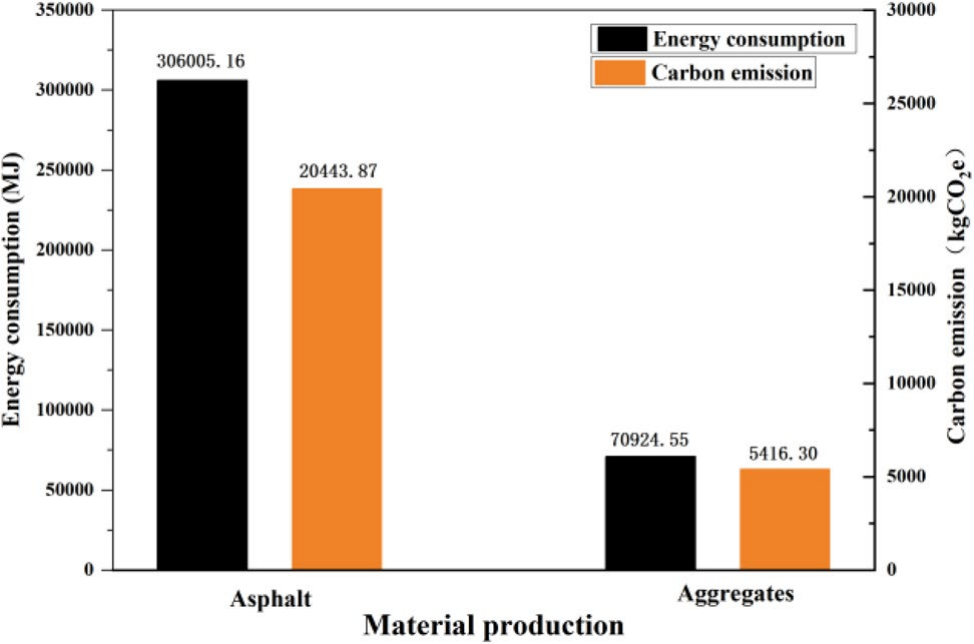
Energy consumption and carbon emission in the material production of asphalt mixture (per 1000 m^3^).

In the material production stage, energy consumption and carbon emissions are primarily attributed to the manufacturing processes of asphalt binder and aggregates. The calculated results are presented in Table 6. In Fig. 3, the energy consumption and carbon emissions associated with the production of base asphalt are substantially higher than those of aggregate materials. This disparity underscores the energy-intensive nature of asphalt production, which involves complex refining and processing of petroleum derivatives. In contrast, aggregate production, encompassing extraction, crushing, and basic processing, entails comparatively lower energy input and associated emissions.

**Table 6:** Energy consumption and carbon emission in material production stage.

Type of materials	Usage (t)	Energy consumption per unit mass (MJ/t)	Energy consumption (MJ)	Carbon emission factor (kgCO_2_e/t)	Carbon emissions (kgCO_2_e)
Asphalt	108.10	2830.76	306,005.16	189.12	20,443.87
Aggregates	2228.93	31.82	70,924.55	2.43	5416.30
The total	2337.03	/	376,929.71	/	25,860.17

The energy consumption associated with material transportation refers to the energy expended in transporting construction materials from the production or processing facility to the asphalt mixing plant. This energy consumption is influenced by both the mode of transportation and the haul distance. In this study, the energy consumption and corresponding carbon emissions are calculated for a scenario involving 30-ton diesel-powered trucks transporting materials over a distance of 50 km by road, as detailed in Table 7. Due to the substantially larger volume of aggregate required compared to base asphalt and additives, the energy consumption and carbon emissions associated with aggregate transportation are markedly higher than those of the other two materials during this stage.

**Table 7:** Energy consumption and carbon emission in material transportation stage.

Type of materials	Usage (t)	Energy consumption per unit mass (MJ/t)	Energy consumption (MJ)	Transportation carbon emission factor (kgCO_2_e/t)	Transportation carbon emissions (kgCO_2_e)
Asphalt	108.10	40.20	4345.62	3.74	404.29
Additives	12.97	40.20	521.39	3.74	48.51
Aggregates	2228.93	40.20	89,602.99	3.74	8336.20
The total	2350.00	/	94,470 .00	/	8789 .00

### Mixture production and transportation stages

The energy consumption during the asphalt mixture production stage primarily arises from the operation of equipment such as loaders, mixing units, and ancillary systems. Among these, the mixing unit accounts for the majority of energy use, as it is responsible for heating asphalt binder and aggregates, as well as blending the constituent materials. However, due to the specific properties of materials used in CMA, the aggregates do not require high-temperature heating during mixing. Instead, they can be combined with the binder either in a mixing drum at ambient temperature or through manual mixing processes. In this study, empirical production data were obtained from a small-scale batch-type mixing plant operating under intermittent conditions, thereby enabling a more accurate representation of energy consumption characteristics in CMA production.

Based on a production schedule of one 8-h shift yielding 300t of asphalt mixture per shift, the total output of 2,350t requires approximately 7.8 shifts. The corresponding energy consumption and carbon emissions for the mixture production stage are summarized in Table 8. The results indicate that energy consumption and carbon emissions associated with modified asphalt heating constitute the dominant component of this stage, accounting for approximately 3.7 times the emissions generated by the mixing process itself. This highlights the significant environmental burden imposed by the thermal energy required for heating.

**Table 8:** Energy consumption and carbon emission in mixture production stage.

Types	Shifts	Electricity consumption per unit shift (kw·h)/shift	Electricity consumption (kw·h)	electrical power Calorific value [MJ/ (kw·h)]	Energy consumption (MJ)	Carbon emission factor [kgCO_2_e/ (kw·h)]	Carbon emissions (kgCO_2_e)
Asphalt and additive heating	7.8	300.00	2340.00	3.60	8424.00	0.5271	1233.41
Agitation	7.8	80.00	624.00	3.60	2246.40	0.5271	328.91
The total	/	/	2964.00	/	10,670.40	/	1562.32

During the transportation stage, the asphalt mixture is delivered using 15-ton tipper trucks. Based on the required output, the transportation operation spans approximately 2.7 shifts, with each shift consuming 67.89 kg of diesel fuel. The number of shifts for transportation (2.7) was calculated based on the total quantity of mixture (2,350 t), the capacity of the 15-t tipper trucks, and the average fuel consumption data per shift obtained from the plant’s operational records.

The resulting energy consumption and associated carbon emissions are calculated and presented in Table 9. These values reflect the environmental impact of mixture transport and contribute to the overall assessment of asphalt pavement construction.

**Table 9:** Energy consumption and carbon emissions in the mixture transportation stage.

Material type	Shifts	Fuel consumption per unit shift (kg/shift)	Total fuel consumption (kg)	Calorific value of diesel fuel (MJ/kg)	Energy consumption (MJ)	Carbon emission factor (kgCO_2_e/kg)	Carbon emissions (kgCO_2_e)
asphalt mixture	2.7	67.89	178.20	42.65	7681.93	3.14	559.54

### Pavement construction stage

Unlike HMA, which requires intensive compaction due to its thermally activated strength development, CMA achieves strength through curing and binder setting at ambient temperatures. As a result, CMA requires only light wheel roller compaction, eliminating the need for heavy pneumatic tire rollers. In some cases, road-moving vehicles can be utilized to achieve the necessary compaction. The energy consumption and carbon emissions associated with this stage are calculated and summarized in Table 10.

**Table 10:** Energy consumption and carbon emissions during the pavement construction stage.

Type of machinery	Shifts	Fuel consumption per unit shift (kg/shift)	Total fuel consumption (kg)	Calorific value of diesel fuel (MJ/kg)	Energy consumption (MJ)	Carbon emission factor (kgCO_2_e /kg)	Carbon emissions (kgCO_2_e)
12.5m paver	1.8	136.41	245.54	42.65	10,472.20	3.14	771.00
6–8t light wheel roller	3.6	54.86	197.50	42.65	8423.20	3.14	620.15
12–15t light wheel roller	3.6	80.92	291.31	42.65	12,424.46	3.14	914.71
(grand) total	/	/	734.35	/	31,319.86	/	2305.86

## Discussion

### Comparison with other studies

To assess the energy-saving and emission-reduction performance of the new type of CMA, a comparative analysis is conducted against conventional HMA and conventional CMA. To ensure consistency and minimize deviations resulting from methodological differences or parameter selection, this study adopts the material quantities for HMA and conventional CMA based on the reference study^15^, as detailed in Table 11. Specifically, the energy consumption and carbon emissions for the mixing stage of HMA and conventional CMA are derived directly from the original literature. For all other stages, energy consumption and emissions are calculated using the same methodology. To facilitate comparison with other research, the results are normalized to reflect energy consumption and carbon emissions per ton of asphalt mixture, as shown in Table 12.

**Table 11:** Amount of materials required for different types of asphalt mixtures.(per 1000 m^3^).

Type of mixture	Volume of mixture (m^3^)	Asphalt (t)	Additives (t)	Aggregate (t)	Total mixture(t)
HMA	1000	116.00	/	2418.00	2534.00
CMA	1000	97.00	/	2300.00	2397.00
New-CMA	1000	108.10	12.97	2228.93	2350.00

**Table 12:** Energy consumption and carbon emissions of different types of per ton asphalt mixtures.

Type of mixture	Material production		Material transportation		Mixture production		mixture transportation		construction process		The total	
	Energy consumption (MJ/t)	Carbon emissions (kgCO_2_e/t)	Energy consumption (MJ/t)	Carbon emissions (kgCO_2_e/t)	Energy consumption (MJ/t)	Carbon emissions (kgCO_2_e/t)	Energy consumption (MJ/t)	Carbon emissions (kgCO_2_e/t)	Energy consumption (MJ/t)	Carbon emissions (kgCO_2_e/t)	Energy consumption (MJ/t)	Carbon emissions (kgCO_2_e/t)
HMA	166.11	11.40	41.75	3.88	182.88	10.01	3.39	0.25	14.69	1.08	408.82	26.62
CMA	157.60	11.03	39.49	3.67	22.82	2.01	3.21	0.24	13.15	0.97	236.27	17.91
New-CMA	154.48	10.60	38.72	3.60	4.37	0.64	3.15	0.23	13.15	0.97	213.86	16.04

The energy consumption of different types of asphalt mixtures reveals that HMA exhibits significantly higher energy demand compared to CMA. The energy savings of New-CMA relative to HMA are largely attributable to the elimination of aggregate heating. In HMA production, aggregates must be heated to 150–180°C to remove moisture and ensure adequate coating by the asphalt binder. In contrast, New-CMA requires only the asphalt binder to be heated (130–160°C), while aggregates remain at ambient temperature. In this study, the energy consumption per ton of asphalt mixture is calculated as 408.82 MJ/t for HMA, 236.27 MJ/t for conventional CMA, and 213.86 MJ/t for the New-CMA. Compared to HMA, the New-CMA achieves an energy reduction of 194.95 MJ/t, representing a 48% decrease. When compared with conventional CMA, New-CMA shows a reduction of 22.41 MJ/t, equivalent to a 9% improvement.

These findings are consistent with previous studies on cold mix asphalt technologies. Meena et al.^31^ conducted a comparative life cycle analysis of cold mix and foamed mix asphalt, evaluating energy consumption and carbon emissions in the cradle-to-gate stages. Their study demonstrated that cold mix technologies significantly reduce environmental impacts compared to hot mix asphalt due to the elimination of aggregate heating. A comprehensive review by Malik et al.^32^ synthesized findings from over 160 studies and reported that CMA reduces energy consumption by 35–50% and carbon emissions by 40–60% compared to HMA through ambient-temperature production with emulsified or cutback binders. The results from the present study fall within this range, with New-CMA achieving a 48% energy reduction and a 40% carbon reduction.

In terms of carbon emissions, the results of this study indicate that HMA produces 26.62 kg CO₂ per ton of mixture, whereas conventional CMA emits 17.91 kg CO₂/t, and the New-CMA emits 16.04 kg CO₂/t. Compared to HMA, New-CMA achieves a carbon reduction of 10.58 kg CO₂/t, representing a 40% decrease. When compared to conventional CMA, the reduction is 1.87 kg CO₂/t, equivalent to a 10% reduction.

These carbon emission reductions are consistent with findings from existing literature. A life cycle assessment of foamed asphalt cold recycled mixtures was conducted by Deng et al.^33^ using the IPCC carbon emission factor method, analyzing four stages: raw material production, mixing, transportation, and construction. Their study found that compared to traditional HMA, the cold recycling technology reduces energy consumption by 51.92% and CO₂e emissions by 34.06%. Gruber and Hofko^25^ assessed the ecological impacts of different asphalt mix designs following a cradle-to-gate approach. Their analysis of production data from an asphalt mixing plant confirmed that cold recycled asphalt pavements contribute to lower global warming potential compared to conventional HMA. A quantitative LCA study on modified recycled asphalt mixtures further identified that the raw material acquisition stage is the dominant contributor to environmental impacts, accounting for over 50% of each major impact category, indicating that utilizing cold recycling technology and increasing the use of recycled materials are efficient ways to promote energy conservation and emission reduction.

It is evident from these studies that cold mix asphalt technologies significantly lower energy consumption and CO₂ emissions—a trend consistent with the findings of this research. It should be noted, however, that many of the referenced studies focused solely on emissions from the mixing stage, whereas the present study adopts a more comprehensive approach covering five key stages (material production, transportation, mixture production, mixture transportation, and pavement construction). As a result, the reported emission values here are comparatively higher, reflecting a more complete assessment of the asphalt mixture production process.

### Comparative analysis of three mixture types

**Fig. 4:**
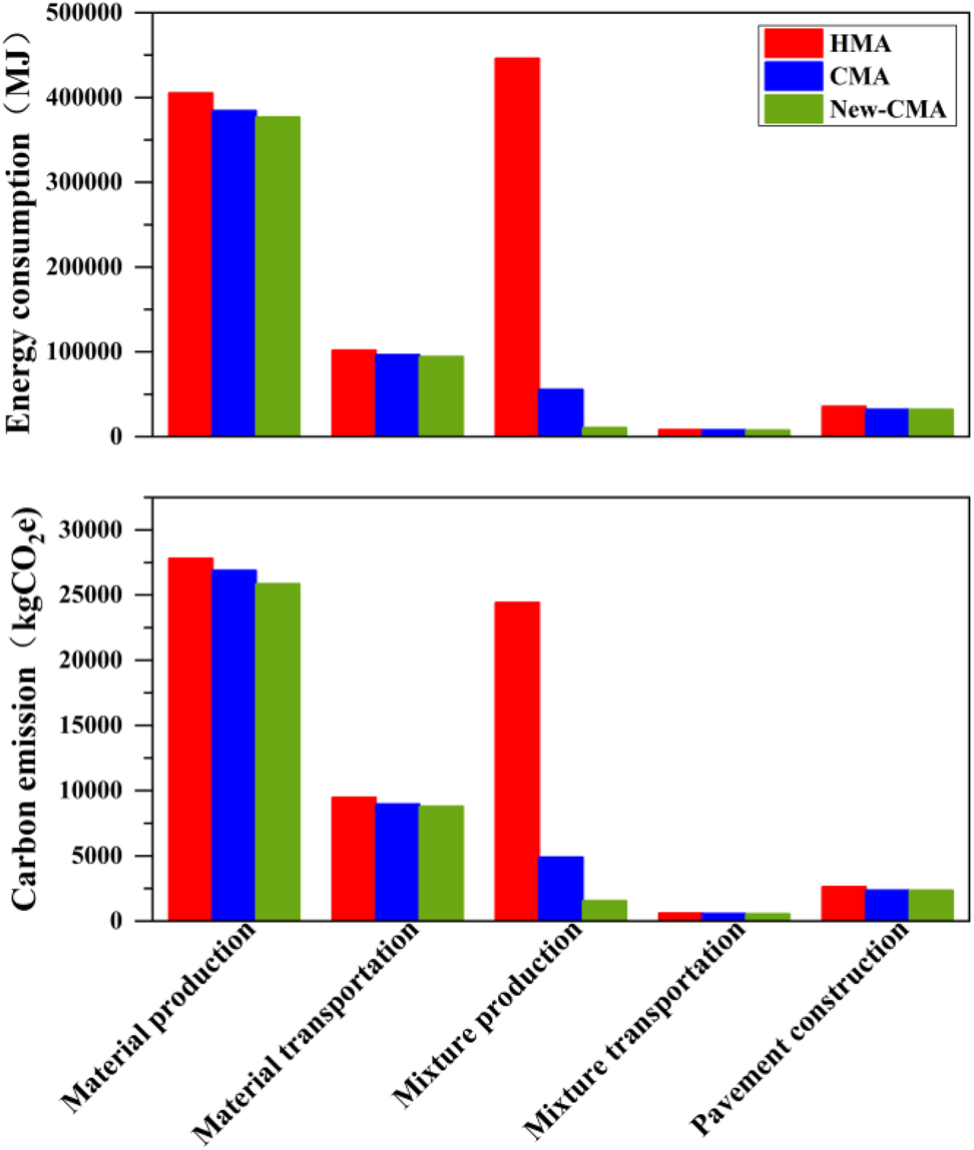
Energy consumption and carbon emission of different types of asphalt mixture (per 1000 m^3^).

The energy consumption and carbon emissions associated with each stage for the three types of asphalt mixtures are illustrated in Fig. 4. With respect to energy consumption, HMA exhibits the highest values during both the material production stage and the mixture production stage, reaching 166.11 MJ/t and 182.88 MJ/t, respectively. In contrast, conventional CMA demonstrates a markedly lower overall energy demand compared to HMA, particularly during the mixture production stage, where its energy consumption is only 12.5% of that observed for HMA. Among the three, the New-CMA shows the most favorable performance in terms of energy efficiency. In the mixture production stage, its energy consumption is merely 2.4% of that of HMA, underscoring the benefits of its optimized process design and/or material composition. These results highlight the significant potential of New-CMA to reduce energy demand in asphalt pavement construction through innovation in low-temperature mixing technologies.

In terms of carbon emissions, the distribution pattern among the three asphalt mixtures closely was similar to the trends observed in energy consumption. Specifically, the carbon emissions of the New-CMA during the mixture production stage are significantly lower than those of both HMA and conventional CMA, amounting to only 6.4% of HMA’s emissions. This substantial reduction underscores the environmental advantages of eliminating high-temperature heating processes in CMA technologies.

Previous studies have consistently identified aggregate drying and heating as the most carbon-intensive operations in mixture production, particularly for HMA and WMA. For instance, Peng et al.^34^ estimated that approximately 90% of total carbon emissions in mixtureproduction are attributable to aggregate and binder heating, as well as the mixing process itself. Li et al.^35^ estimated that drying and heating aggregates contribute approximately 94% to 97% of total CO₂ emissions from the thermal energy used for heating both aggregates and bitumen at asphalt plants. Similarly, Ferrotti et al.^22^ reported that this process accounts for 92% to 96% of total CO₂ emissions across various mixture types and production technologies. Wang et al.^36^ further confirmed that carbon emissions from the aggregate heating stage can reach 96%–98% of the total. These findings reinforce the conclusion that the substantial reduction in carbon emissions observed in New-CMA is primarily due to the elimination of thermal energy requirements for aggregate processing, thereby confirming its potential as a sustainable alternative in pavement construction.

**Fig. 5:**
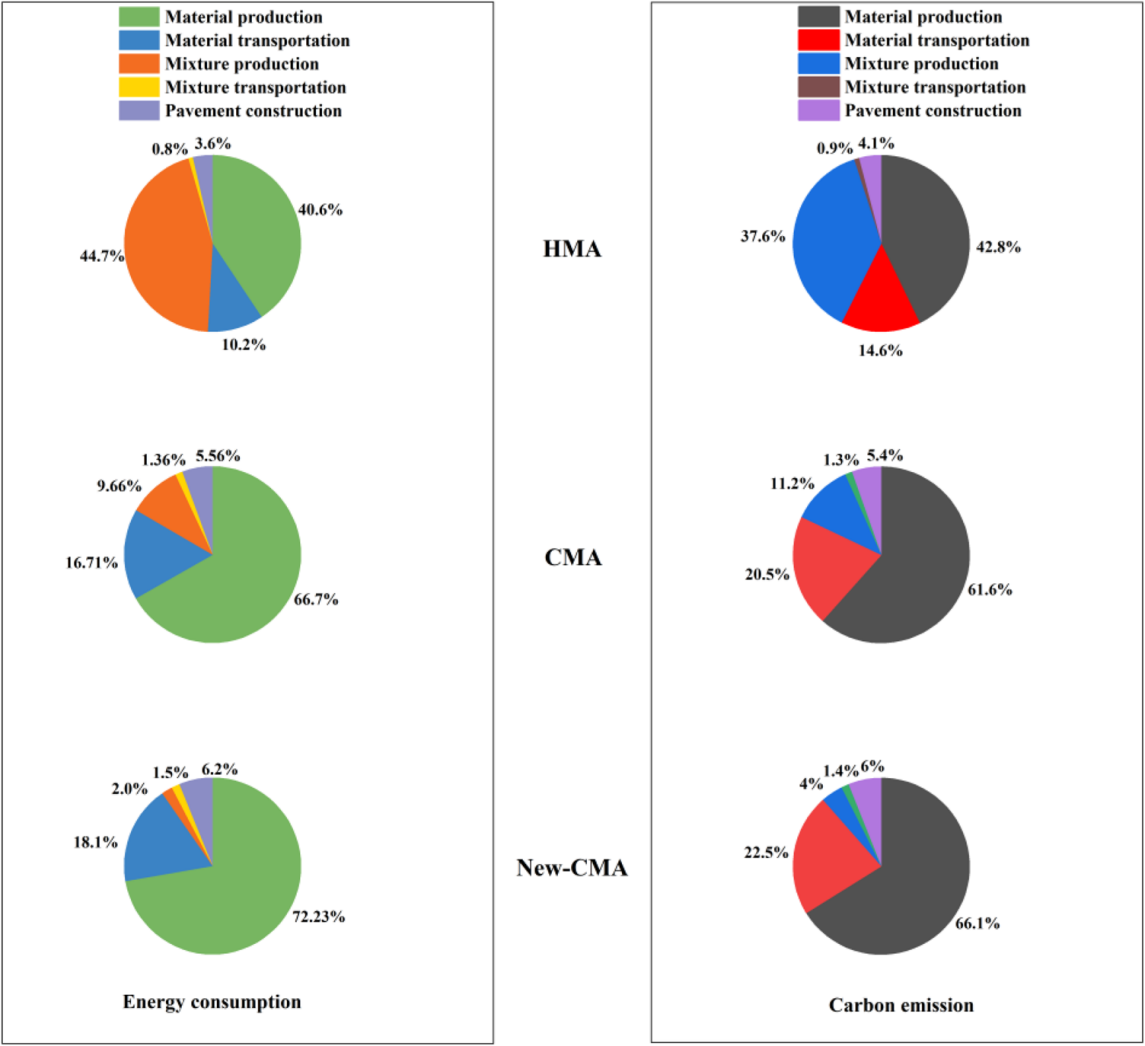
Energy consumption and carbon emission share of different types of asphalt mixture.(per 1000 m^3^).

In Fig. 5, the material production and mixture production stages account for the largest proportions of total energy consumption in the production process of HMA, comprising 40.63% and 44.73%, respectively. In contrast, energy consumption is predominantly concentrated in the material production and transportation stages for CMA, while the energy share of mixture production is substantially reduced. Specifically, mixture production accounts for only 9.66% of total energy consumption in conventional CMA and as little as 2% in the New-CMA. A similar distribution pattern is observed for carbon emissions. HMA exhibits the highest emissions during material production and mixture production, with shares of 42.8% and 37.6%, respectively. Conversely, in CMA, the contribution of mixture production to total carbon emissions is markedly lower, representing 11.2% for conventional CMA and only 4% for New-CMA.

These results demonstrate that energy consumption and carbon emissions during the mixture production stage constitute a critical portion of the total environmental impact across mixtureproduction processes. Unlike HMA, CMA eliminates the need for heating aggregates or mixtures during mixing, thereby substantially reducing energy consumption and associated emissions at this stage. The New-CMA achieves further reductions compared to conventional CMA, attributable to the optimized additive formulation developed in this study, which enables mixing at ambient or even lower temperatures. Additionally, the mixing tower used the electricity as the primary energy source for the mixing process in this study, making more efficient relative to traditional fuel-powered equipment. Therefore, lower energy consumption was observed in New-CMA. Almeida et al.^37^ emphasize that significant reductions in energy demand and carbon emissions can be realized through the use of dry aggregates and alternative energy sources such as natural gas, highlighting the potential for further improvements by optimizing construction methods and energy inputs.

### Detailed breakdown for new-CMA

To provide a more comprehensive interpretation, the energy consumption and carbon emissions for New-CMA are broken down by stage, material type, and energy source. As shown in Table 13, the material production stage contributes 72.4% of total energy consumption (376,929.71 MJ) and approximately 66% of total carbon emissions (25,860.17 kgCO₂e). Within this stage, asphalt production is the dominant contributor, accounting for 81.2% of material production energy (306,005.16 MJ) and 79.0% of material production emissions (20,443.87 kgCO₂e). This disparity reflects the energy-intensive nature of bitumen refining, which involves complex processes such as distillation, cracking, and blending. In contrast, aggregate production—consisting of extraction, crushing, and screening—requires significantly less energy per unit mass. To further reduce the environmental footprint of New-CMA, priority should be given to sourcing low-carbon asphalt binders (e.g., bio-based or recycled asphalt) or improving the energy efficiency of asphalt refining. Aggregates, despite their lower per-unit impact, still contribute notably due to their large mass fraction.

**Table 13:** Detailed breakdown of energy consumption and carbon emissions for New-CMA (per 1000 m^3^).

Stage	Material/Activity	Energy source	Energy (MJ)	CO₂e (kg)
Material production	Asphalt	Embedded	306,005.16	20,443.87
	Aggregates	Embedded	70,924.55	5,416.30
Material transportation	Asphalt	Diesel	4,345.62	404.29
	Additive	Diesel	521.39	48.51
	Aggregates	Diesel	89,602.99	8,336.20
Mixture production	Binder + additive heating	Electricity	8,424.00	1,233.41
	Mixing operations	Electricity	2,246.40	328.91
Mixture transportation	Mixture transport	Diesel	7,681.93	559.54
Pavement construction	Paver and rollers	Diesel	31,319.86	2,305.86
Total			521,071.90	39,076.89

The material transportation stage accounts for 18.1% of total energy consumption (94,470.00 MJ) and 22.5% of total carbon emissions (8,789.00 kgCO₂e). Within this stage, aggregate transportation dominates, representing 94.9% of both energy and emissions. This is due to the large mass of aggregates required (2,228.93 t per 1000 m^3^, accounting for 94.8% of total material mass). While the per-ton energy intensity of aggregate transport (40.20 MJ/t) is the same as for asphalt and additive, the sheer volume of aggregates makes transportation a critical factor. Locating aggregate sources closer to the mixing plant could yield substantial reductions in overall environmental impact.

The mixture production stage accounts for only 2.0% of total energy consumption (10,670.40 MJ) and 4.0% of total carbon emissions (1,562.32 kgCO₂e). This is a dramatic reduction compared to HMA, where the mixture production stage accounts for 44.7% of total energy consumption. Within this stage, binder heating is the dominant contributor, accounting for 78.9% of electricity consumption (8,424 MJ) and 78.9% of associated emissions (1,233.41 kgCO₂e). Mixing operations account for the remaining 21.1%. The low contribution of the mixture production stage validates the core environmental advantage of New-CMA: the elimination of aggregate heating. Further reductions could be achieved by lowering the binder heating temperature (currently 130–160°C) or developing binders with even lower viscosity at ambient temperatures. However, given that this stage already contributes minimally, such improvements would have limited additional impact on the overall footprint.

The mixture transportation stage contributes 1.5% of total energy consumption (7,681.93 MJ) and 1.4% of total carbon emissions (559.54 kgCO₂e). This relatively low contribution reflects the shorter transport distance (20 km) compared to material transport (50 km). The pavement construction stage contributes 6.0% of total energy consumption (31,319.86 MJ) and 5.9% of total carbon emissions (2,305.86 kgCO₂e). This stage includes paver and roller operations. While the construction stage is not the primary focus of emission reduction strategies, optimizing equipment operation (e.g., reducing idle time, using fuel-efficient machinery) could yield moderate benefits.

The breakdown clearly demonstrates that the environmental advantages of New-CMA are primarily derived from the elimination of aggregate heating in the mixture production stage, which reduces its contribution from 44.7% (in HMA) to just 2.0% of total energy consumption. This shifts the dominant impact to the material production and transportation stages, where further improvements can be targeted.

### Sensitivity and uncertainty analysis

To assess the robustness of the results and the influence of key parameters, a sensitivity analysis was conducted on the New-CMA. The parameters selected for analysis include: (1) transportation distance for materials and mixture; (2) electricity emission factor; (3) diesel emission factor; (4) additive production energy intensity; and (5) asphalt production energy intensity. Each parameter was varied by ± 20% (or ± 50% for transportation distance), and the resulting changes in total energy consumption and carbon emissions were calculated. The results are summarized in Table 14.

**Table 14:** Sensitivity analysis results for New-CMA (per 1000 m^3^).

Parameter	Variation	Change in energy (%)	Change in CO₂e (%)
Material transportation distance	+ 50%	+ 9.1	+ 11.2
	−50%	−9.1	−11.2
Mixture transportation distance	+ 50%	+ 0.7	+ 0.7
	−50%	−0.7	−0.7
Electricity emission factor	+ 20%	0	+ 0.8
	−20%	0	−0.8
Diesel emission factor	+ 20%	0	+ 5.0
	−20%	0	−5.0
Additive production energy	+ 2830 MJ/t	+ 7.0	+ 6.3
Asphalt production energy	+ 20%	+ 11.7	+ 10.5
	−20%	−11.7	−10.5

The analysis reveals that asphalt production energy intensity and material transportation distance are the most influential parameters, with variations of ± 20% in asphalt production energy resulting in approximately ± 11% changes in total energy consumption. The additive production stage is not quantitatively included in this analysis due to its minimal mass contribution (0.55% of total mixture) and limited data availability. Even under the conservative assumption that additive production has the same energy intensity as asphalt (2830 MJ/t), its contribution would be approximately 36,700 MJ per 1000 m^3^ of mixture, or only 7% of total energy consumption. In practice, the actual contribution is expected to be lower.

Regarding uncertainty, the primary data used in this study (material quantities, fuel consumption, electricity consumption) were collected directly from the mixing plant over 15 production shifts, providing a reliable basis for the calculations. Secondary data, such as material production energy intensities and emission factors, were sourced from the Chinese Life Cycle Database (CLCD) and IPCC guidelines, which are widely accepted in the field. Where local data were available (e.g., electricity emission factor for Guizhou region), they were used to improve representativeness.

Direct CO₂ emissions from the mixture production stage are not separately calculated in this study. This is justified by the ambient-temperature nature of cold mix asphalt technology. Unlike HMA, which requires high-temperature heating of both aggregates and binder (typically 150–180 °C) and generates substantial direct combustion emissions from fuel-fired dryers and heating systems, the New-CMA process eliminates aggregate heating entirely. The mixing tower at the investigated plant operates on electricity, and no on-site fuel combustion occurs during mixing. Furthermore, the mixture is paved at ambient temperature, which further reduces potential emissions. Consequently, any direct CO₂ emissions associated with the mixture production stage are minimal and are fully captured through the electricity consumption emission factor used in this study. Therefore, the exclusion of direct CO₂ emissions does not affect the comparative conclusion that New-CMA achieves significant emission reductions; rather, it may result in a conservative estimate of the emission savings.

Overall, the sensitivity analysis indicates that the main conclusions—specifically that New-CMA achieves substantial energy and emission reductions compared to HMA—remain robust under reasonable variations in key parameters.

### Limitations regarding functional equivalence

This study assumes functional equivalence between HMA, conventional CMA, and New-CMA based on the same volume of pavement (1000 m^3^) for low-to-medium traffic applications. However, it is important to note that the service life and structural performance of the three mixtures may differ. While the New-CMA has been applied in local road surface repairs and paving with satisfactory early performance, long-term durability data are not yet available. Differences in service life would affect the comparative environmental performance over the full pavement life cycle. Future research should incorporate long-term performance monitoring and life cycle assessment that accounts for potential differences in durability and maintenance requirements.

## Conclusions

This study presents a comparative analysis of the energy consumption and carbon emissions associated with the production and construction of the New-CMA, conventional CMA, and HMA. Based on the quantitative assessment across five key stages, the key conclusions are as follows:

### Significant energy savings and emission reductions

Within the defined system boundary, the New-CMA technology exhibits significant environmental advantages in both energy consumption and carbon emissions reduction. Compared with HMA, New-CMA reduces energy consumption by 48% and carbon emissions by 40%. These reductions are primarily attributed to the elimination of aggregate heating. However, it should be noted that the use stage, maintenance activities, and end-of-life treatment were not included in this analysis; therefore, the reported savings represent only the production and construction phases.

### The mixing stage is the critical point for emission mitigation

The mixing stage was identified as a key contributor to the overall energy and emission profile, particularly for HMA, where it accounts for 44.73% of energy consumption and a similarly high proportion of emissions. In contrast, the mixing stage of New-CMA contributes only 2.04% of total energy consumption. This dramatic reduction is achieved by eliminating the need for high-temperature heating, making CMA, especially the optimized New-CMA, an optional solution in advancing low-carbon pavement technologies.

### Importance of a comprehensive production-stage perspective

By adopting a systematic assessment framework, this study quantifies energy consumption and carbon emissions across five key stages from material production to pavement construction, including material production, transportation, mixture production, and construction. The analysis reveals that CMA not only achieves substantial emission reductions during the mixing stage but also contributes to further environmental benefits by minimizing the use of construction machinery, such as eliminating the need for pneumatic tire rollers. This comprehensive evaluation confirms that CMA, and particularly New-CMA, offers a promising pathway toward more sustainable pavement practices.

Although this study has achieved some results, the following limitations still exist: (1) Limitations of data sources: Some of the energy consumption and carbon emission factors refer to common databases or literature data, which may deviate from the actual production conditions, and need to be further verified by combining with field measurement data in the future. (2) Limited system boundary: The system boundary of this study does not cover the entire pavement life cycle. This study does not consider the performance degradation of asphalt mixtures during the use stage, nor does it account for energy consumption and direct emissions during maintenance and waste treatment. Further studies could extend the system boundary to include these phases for a more comprehensive environmental assessment. (3)Functional equivalence assumption: This study assumes that HMA, conventional CMA, and New-CMA serve the same function (pavement construction) based on volume. However, differences in service life, durability, and maintenance requirements have not been considered. The environmental advantages reported for New-CMA are based on production and construction stages only and do not account for potential differences in long-term performance.

In conclusion, New-CMA demonstrates excellent potential as a high-efficiency, low-carbon pavement material. These characteristics make it a promising solution for sustainable pavement practices in both urban maintenance and new road construction. Continued innovation and lifecycle-based evaluation will be essential to fully realize its environmental and engineering benefits.

## Data Availability

The data presented in this study are available on request from the corresponding author.
